# Quality assurance tests for the Gamma Knife^®^ Icon™ image guidance system

**DOI:** 10.1002/acm2.12417

**Published:** 2018-08-03

**Authors:** Ismail AlDahlawi, Dheerendra Prasad, Matthew B. Podgorsak

**Affiliations:** ^1^ Department of Radiation Medicine Roswell Park Comprehensive Cancer Center Buffalo NY USA; ^2^ Department of Neurosurgery Roswell Park Comprehensive Cancer Center Buffalo NY USA; ^3^ Medical Physics Program Jacobs School of Medicine and Biomedical Sciences State University of New York at Buffalo Buffalo NY USA; ^4^ Department of Radiation Oncology Jacobs School of Medicine and Biomedical Sciences State University of New York at Buffalo Buffalo NY USA; ^5^ Department of Neurosurgery Jacobs School of Medicine and Biomedical Sciences State University of New York at Buffalo Buffalo NY USA; ^6^ Department of Radiation Oncology King Fahad Specialist Hospital‐Dammam Dammam Saudi Arabia

**Keywords:** Gamma knife, Icon, motion management, QA tool, stereotactic radiosurgery

## Abstract

**Introduction:**

The Gamma Knife^®^ Icon™ comes with an image guidance system for tracking patient motion and correcting for inter‐ and intrafractional shifts, mainly used with frameless thermoplastic immobilization. The system consists of a cone‐beam CT (CBCT) and a couch‐mounted infrared camera (IFMM). We report our quality assurance program for Icon's image guidance system.

**Methods:**

The manufacturer‐provided tool is used for daily checks of CBCT positional precision. Catphan^®^ phantom is used for monthly checks of CBCT image qualities (uniformity, contrast to noise ratio (CNR), and spatial resolution) for the two acquisition presets (low‐dose and high‐quality presets). On a semi‐annual schedule, we use a frame tool to check the agreement of CBCT‐based and Frame‐based stereotactic space coordinates by comparing the locations of five attached ball bearings in CT‐sim scans (Frame‐based coordinates determination) and in Icon's CBCT scans. On an annual basis, the accuracy of IFMM, image registration, and delivery‐after‐shift are tested using a translational stage. A weighted CT dose index is measured annually with a pencil chamber in CTDI head phantom.

**Results:**

The CBCT precision check: 0.12 ± 0.04 mm (maximum deviations average). CBCT image quality: spatial resolution range: [6,7] lp/cm (low), and [7,8] lp/cm (high); uniformity: 12.82 ± 0.69% (low), and 13.01 ± 0.69% (high); CNR: 1.07 ± 0.08 (low), and 1.69 ± 0.10 (high). Agreement of CBCT‐based with Frame‐based stereotactic coordinates range: [0.33, 0.66] mm. Accuracy of IFMM: 0.00 ± 0.12 mm (average) with 0.27 mm (max.); image registration: 0.03 ± 0.06 mm (average) with 0.23 mm (max.); and delivery‐after‐shift: 0.24 ± 0.09 mm (average) with 0.42 mm (max.). CTDI_w_: 2.3 mGy (low), and 5.7 mGy (high).

**Conclusions:**

The manufacturer‐required QA checks together with additional user‐defined checks are an important combination for a robust quality assurance program ensuring the safe use of Gamma Knife^®^ Icon™'s image guidance and motion management features.

## 1 INTRODUCTION

The high dose of radiation delivered to intracranial lesions in stereotactic radiosurgery (SRS) mandates precise localization. With current available technology, it is possible to localize targets with a noninvasive frameless system with the help of image guidance and motion management tools, as opposed to the traditional invasive frame system fixed to the skull. The Leksell Gamma Knife^®^ Icon™ (Elekta A.B., Stockholm, Sweden) comes equipped with an image guidance system that makes it feasible to use a frameless thermoplastic fixation mask instead of the traditional SRS invasive frame. The Icon comes with a Cone‐beam CT (CBCT) system that can be used for defining the Leksell stereotactic space using imaging without the need for the traditional frame system. CBCT is also used in pretreatment for determining any translational and rotational shifts of the patient skull with respect to the reference CBCT image. For tracking intrafractional motions, the Icon comes with an Intra‐Fraction Motion Management (IFMM) system consisting of an infrared (IR) camera that tracks the movement of a reflective marker, typically placed on the patient's nose, with respect to reference reflective markers permanently attached to the head rest adapter during treatment delivery. The CBCT and IFMM system specifications were described in previous publications.^1^, ^2^

A typical frameless patient treatment workflow starts with planning on nonstereotactic CT or MRI images that provide the needed anatomical information for treatment planning. A reference CBCT on the Icon system is taken with the patient head laying on a custom pillow and immobilized with the frameless thermoplastic system. This reference CBCT image is used to define the Leksell stereotactic coordinates and is coregistered with the planning CT/MRI image in Leksell GammaPlan (LGP) software (V11.0.2, Elekta Instruments, A.B., Stockholm, Sweden). Prior to treatment delivery, a setup CBCT is taken in the treatment position and is co‐registered with the reference CBCT to determine the interfractional shifts. Any translation or rotation differences between the setup CBCT and reference CBCT are calculated by the LGP software and an optimum translational couch shift is proposed. A new dose distribution plan is shown for physician approval taking into account the change in plan due to rotational/translational shifts. During the treatment delivery, the intrafractional motion is determined by tracking a reflector marker placed on the patient nose detected by the couch‐mounted IR camera. A displacement of this reflector above a threshold, that can be set from 0.5 to 3 mm, triggers an automatic delivery stop (in active mode). Intrafractional setup CBCTs can be acquired and coregistered with the reference CBCT if the marker displacement did not return back below the threshold, and the process of coregistration with the reference CBCT and plan adaptive is repeated.^3^

In this study, we share our QA program for testing the image guidance features of the Gamma Knife Icon.

## 2 MATERIALS AND METHODS

### 2.A Manufacturer's required CBCT tests

The manufacturer requires two tests to be performed routinely by the user for the Icon's CBCT system: (a) CBCT Precision Test and (b) CBCT Image Quality Test.

#### 2.A.1 CBCT precision test

This is a console‐programmed test to check the CBCT positional precision. The user scans the manufacturer‐provided tool (QA Tool Plus) and the test algorithm detects the coordinates of four fiducial markers on the tool. The algorithm compares the coordinates with baseline values that were determined by a manufacturer's service calibration tool. The test algorithm also calculates the CBCT image volume and compares points with baselines to report the maximum deviation value.^4^ This test is required by the manufacturer to be performed once a month but it is recommended to be carried out daily. We report our daily maximum deviation values for a 2‐yr period in this work.

#### 2.A.2 CBCT image quality tests

The second test required by the manufacturer is to check image qualities for the two scanning presets available on the Icon: the low‐dose preset of nominal CTDI = 2.5 mGy and the high‐quality preset of nominal CTDI = 6.3 mGy. The CBCT image quality test is required by the manufacturer to be performed on a semi‐annual schedule, with the recommendations to be performed monthly. The Catphan^®^ 503 phantom (The Phantom Laboratory, Salem, NY) is CBCT scanned for this test and different modules within the phantom are utilized to check for spatial resolution; contrast to noise ratio (CNR); and image uniformity.

The spatial resolution was determined by an observer finding the highest numbered line pair that can be seen in a CBCT image of the phantom.

The CNR was calculated from an image of a polystyrene and LDPE inserts using the equation:

(1) $$CNR=\frac{{\overset{¯}{I}}_{\mathrm{PS}}-{\overset{¯}{I}}_{\mathrm{LDPE}}}{\sqrt{\sigma_{\mathrm{PS}}^{2}+\sigma_{\mathrm{LDPE}}^{2}}}$$

where ${\overset{¯}{I}}_{\mathrm{PS}}$ and ${\overset{¯}{I}}_{\mathrm{LDPE}}$ indicate the mean pixel values using a 5‐mm square image probe for the polystyrene and LDPE, respectively; and *σ*_*PS*_ and *σ*_*LDPE*_ indicate the standard deviation values for the same inserts.

For the uniformity test, five readings were taken at the center, 12, 3, 6, and 9 o'clock positions of an image of a homogeneous section of the Catphan 503 phantom using a 10‐mm square image probe. The uniformity was calculated using the formula:

(2) $$uniformity=\frac{{\overset{¯}{I}}_{\max}-{\overset{¯}{I}}_{\min}}{{\overset{¯}{I}}_{\max}+1000}\times 100\%$$

where *Ī_max_* is maximum mean pixel value, and *Ī_min_*is the minimum mean pixel value of the five readings.^5^

We report our 2‐yr results of the manufacturer monthly image quality tests in this work.

### 2.B User‐defined image guidance tests

In our clinic, we developed additional independent tests to check the agreement of CBCT‐based stereotactic space with Frame‐based stereotactic space, the accuracies of the IFMM system, registration algorithm, and delivery‐after‐shift, and to monitor the CBCT dose consistency through weighted CT dose index (CTDI) measurements.

#### 2.B.1 Agreement of CBCT‐based stereotactic space with Frame‐based stereotactic space

**Figure 1:**
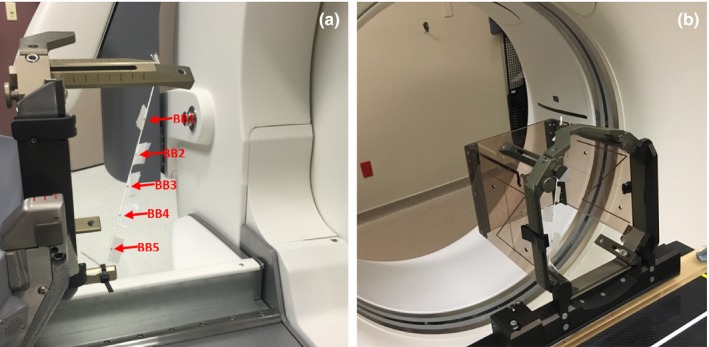
CBCT‐based and Frame‐based stereotactic space agreement tool setup at (a) Gamma Knife Icon for defining the CBCT‐based stereotactic coordinates of five BBs hanged diagonally on the stereotactic frame; and (b) CT‐simulator with the indicator box for defining the Frame‐based stereotactic coordinates for the same stereotactic frame with BBs.

The CBCT system is calibrated to use the Leksell Coordinate System (LCS) for stereotactic space definition. In order to test the agreement between the stereotactic reference CBCT and the gold‐standard Frame‐based definition of Leksell stereotactic space, we used a simple tool consisting of five ball‐bearing (BB) fiducial markers (0.5 mm diameter) attached to a taut string hung diagonally along stereotactic frame posts (Fig. 1). The frame was scanned in our CT simulator (Discovery RT, GE, San Diego, CA, USA) with the standard CT fiducial indicator box. The Frame‐based stereotactic coordinates of each BB were determined in the LGP software. The BB coordinates are then compared with coordinates determined from a CBCT scan of the tool. The magnitude of the three‐dimensional (3D) vector difference (*r*) between the coordinates of Frame‐based and CBCT‐based is calculated as:

(3) $$r=\sqrt{{\left(x_{\mathrm{Frame}}-x_{\mathrm{CBCT}}\right)}^{2}+{\left(y_{\mathrm{Frame}}-y_{\mathrm{CBCT}}\right)}^{2}+{\left(z_{\mathrm{Frame}}-z_{\mathrm{CBCT}}\right)}^{2}}$$

#### 2.B.2 Accuracy tests of IFMM system, registration algorithm, and delivery‐after‐shift

**Figure 2:**
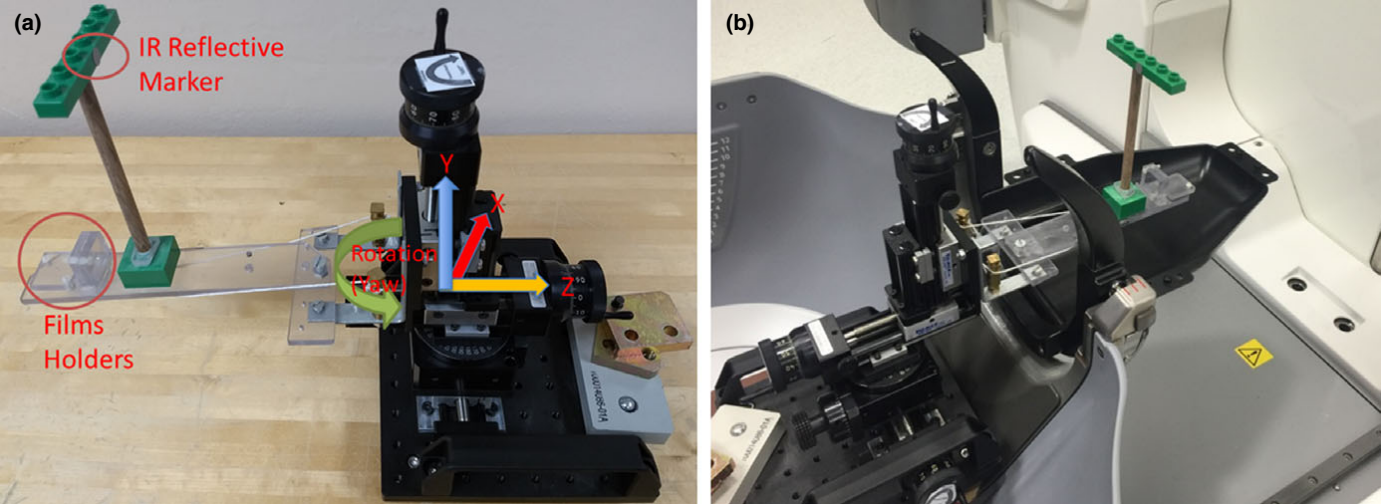
(a) A picture of the linear translational stage tool with the added rotary component. A Lucite platform is attached to the transitional stage with a post with the IR reflective marker to check the IFMM accuracy, and two film holders to check delivery‐after‐shift accuracy; and (b) The same tool setup on Icon's couch. The head mask adapter which holds reference markers is attached to the couch to activate the IFMM and registration with plan adaptive functions in the Icon system.

For these tests, we used a manual linear translational stage (Velmex Inc., Bloomfield, NY) with submillimeter shift accuracy in the three perpendicular planes: *x* (lateral), *y* (vertical), and *z* (longitudinal). A rotary component added to this stage allowed for subdegree rotation along the vertical *y*‐axis (yaw). A Lucite platform is attached to the translational stage and falls in the CBCT field of view when scanning the tool. A post is attached to the platform with an infrared reflector marker for testing the IFMM system movement accuracy. The platform also hosts two film holders that can be embedded with Gafchromic EBT2 films (International Specialty Products, NJ, USA) in two different locations and positions; one being parallel to the couch motion plane (*xz* plane), while the other is perpendicular to the couch plane (*yz* plane) (Fig. 2). The holders have holes for punching the embedded films that are visible in CBCT images and are used for radiation shot placement.

**Figure 3:**
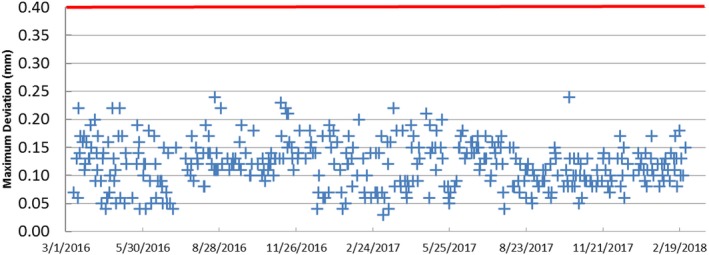
Maximum deviation (mm) in image volume of the daily CBCT precision test over 2‐yr period. The red line represents the manufacturer limit for the test to pass (0.4 mm).

For performing the accuracy tests, a treatment plan was created based on CT simulator images with two shots placed on the film planes just under the visible holes using the smallest collimator (4 mm). A reference CBCT was then taken with the Icon system and coregistered with the planning CT. A shift was introduced with the stage prior to the setup CBCT to mimic an interfractional motion, and the IFMM system was checked for reading accuracy against this shift. A setup CBCT was taken next, and was coregistered with reference CBCT to calculate the shifts with the LGP registration algorithm which was checked against the actual introduced shifts. Two shift scenarios were tested in our work: (a) A small shift scenario (2 mm in *x*, 2 mm in *y*, and 2 mm in *z* directions); (b) A large shift scenario (20 mm in *z*, 5 mm in *x,* and 5 mm in *y* followed by a rotation of 1 degree).

The shots delivery was carried out for the same scenarios described above. The films were scanned with an Epson Perfection V700 flatbed color scanner (Epson America, Long Beach, CA) and processed in MATLAB release 2015b (The MathWorks Inc., Natick, MA) to determine the deviation of radiation shot center from the punched point. These accuracy tests were repeated six times each for establishing baselines.

A second method to check the registration and IFMM accuracy was to apply the rotational and translational shift parameters indicated by the LGP registration algorithm in a rigid transformation matrix formula to calculate the new coordinates of a selected reference point after the shifts. As described by Wright et al., the predicted coordinates (*x*_*b*_ *, y*_*b*_ *, z*_*b*_) can be calculated from the reference coordinates (*x*_*a*_ *, y*_*a*_ *, z*_*a*_) after translational shifts (Δ*x*, Δ*y*, Δ*z*) and rotational shifts around the LCS center (100, 100, 100) as:^3^

(4) $$\left[\begin{array}{c} x_{b}\\ y_{b}\\ z_{b} \end{array}\right]=R\cdot \left[\begin{array}{c} x_{a}-100\\ y_{a}-100\\ z_{a}-100 \end{array}\right]+T$$

where *T* is the translation matrix,

(5) $$T=\left[\begin{array}{c} \Delta x+100\\ \Delta y+100\\ \Delta z+100 \end{array}\right];$$

*R* is the rotation matrix,

(6) $$R=R_{z}\left(\theta_{z}\right)\cdot R_{y}\left(\theta_{y}\right)\cdot R_{x}\left(\theta_{x}\right)$$

with

(7) $$\begin{array}{c} R_{z}\left(\theta_{z}\right)=\left[\begin{array}{ccc} \text{cos}(\theta_{z}) & -\text{sin}(\theta_{z}) & 0\\ \text{sin}(\theta_{z}) & \text{cos}(\theta_{z}) & 0\\ 0 & 0 & 1 \end{array}\right]; \end{array}$$

$\theta_{z}$ being the angle of rotation about the *z*‐axis;

(8) $$R_{y}\left(\theta_{y}\right)=\left[\begin{array}{ccc} \text{cos}(\theta_{y}) & 0 & \text{sin}(\theta_{y})\\ 0 & 1 & 0\\ -\text{sin}(\theta_{y})0 & 0 & \;cos(\theta_{y}) \end{array}\right];$$

$\theta_{y}$ being the angle of rotation about the *y*‐axis; and

(9) $$R_{x}\left(\theta_{x}\right)=\left[\begin{array}{ccc} 1 & 0 & 0\\ 0 & \text{cos}(\theta_{x}) & -\text{sin}(\theta_{x})\\ 0 & \text{sin}(\theta_{x}) & \text{cos}(\theta_{x}) \end{array}\right];$$

$\theta_{z}$ being the angle of rotation about the *x*‐axis.

The calculated coordinates were compared with the actual coordinates of the same point as identified in the treatment console of postshift CBCT images. We tracked two points in our stage tool while applying the large shifts scenario: point A being close to the nose marker, and point B being at the punch hole of the vertical film holder. The 3D vector magnitude of point A (between reference and calculated coordinates) was used to compare and check the IFMM reading accuracy.

#### 2.B.3 CBCT dose constancy test

A weighted CT dose index measurement was performed for both CBCT low‐dose and high‐quality presets using a 10‐cm pencil ionization chamber (Radcal Model 10X6‐3CT, Radcal Corporation, Monrovia, CA) in a CTDI Lucite head phantom. The head phantom was placed on a Styrofoam spacer on the Icon's patient head rest to bring the phantom central in the CBCT field of view. The weighted CTDI (CTDI_w_) was derived from weighted average dose measurements of central and peripheral values according to the formalism described in AAPM Task Group 23 report:^5^

(10) $${\text{CTDI}}_{W}=1/3\;\;\text{CTDI}\left(\text{center}\right)+2/3\;\;\text{CTDI}\left(\text{periphery}\right)$$

We report the results obtained during commissioning and two annual CTDI_w_ measurements for both CBCT presets.

## 3 RESULTS AND DISCUSSION

### 3.A CBCT precision QA test results

The plot in Fig. 3 shows the daily maximum deviation in image volume for the CBCT precision QA test for a 2‐yr period (March 2016–March 2018). The average was 0.12 ± 0.04 mm with a maximum of 0.24 mm. Our results for this test were well below the acceptable limit (<0.4 mm) as established by the manufacturer. We plan to continue performing the CBCT precision test on clinical days (i.e., on days that we use the CBCT for patient treatments) as recommended by the manufacturer.^5^

### 3.B CBCT image quality results

Table 1 summarizes our monthly CBCT image quality results over the 2‐yr period for our Icon and the corresponding manufacturer‐acceptable limits. Our results meet the specifications for the image quality tests, with some exception in the spatial resolution of the low‐dose preset. The results of the spatial resolution test were on the borderline in two nonsequential months (out of the 24 months reported). Considering that the spatial resolution test is a subjective test and the low‐dose preset was used, we find these minor deviations acceptable. The high‐quality preset showed better image quality in terms of spatial resolution and CNR compared to low‐dose preset. However, the uniformity was comparable using either of the two presets. Zeverino et al. reported a similar spatial resolution (7 lp/cm) for both presets, and better image qualities for the high‐quality preset in terms of CNR: 1.2 vs 0.8 (high/low presets); and uniformity: 8.8% vs 9.3% (high/low presets).^2^ Dorenlot and Champoudry used different software (MyQA, IBA Dosimetry, Schwarzenbruck, Germany) with the Catphan 503 phantom to evaluate the image qualities, and reported lower spatial resolution values than ours of 4.9 and 4.8 lp/cm (low/high presets); higher CNR values of 1.41 and 2.24 (low/high presets). As for the uniformity test, it seems they used a different formalism as they reported values of 83.14 and 86.57 (low/high presets). We plan to continue performing the image quality tests on a monthly basis, as recommended by the manufacturer^5^ and AAPM Task Groups 142^7^ and 179,^8^ and adhere to the manufacturer specifications listed in Table 1.

**Table 1:** Summary results of monthly image quality tests (spatial resolutions, CNR, and uniformity) with the manufacturer specification for each test

	[Range] or average ± SD		
	CTDI 2.5 mGy (low‐dose preset)	CTDI 6.3 mGy (high‐quality preset)	Manufacturer specification
Spatial resolution	[6,7] lp/cm	[7,8] lp/cm	Minimum 6 lp/cm
CNR	1.07 ± 0.08	1.69 ± 0.10	>0.5 (CTDI 2.5) >0.8 (CTDI 6.3)
Uniformity	12.84% ± 0.70	13.01% ± 0.69	<14%

### 3.C Agreement of CBCT‐based with Frame‐based stereotactic space results

Table 2 shows the *xyz* coordinates of each BB in Frame‐based stereotactic space and CBCT‐based stereotactic space and their differences. The mean 3D deviation vector (*r*) was 0.5 ± 0.1 mm. In a previous 6‐week study of stability of CBCT coordinates,^1^ we had smaller differences (up to 0.33 ± 0.21 mm in *r*) when comparing daily CBCT coordinates with an average reading of five CT scans (using a different CT‐simulator unit) taken over the 6‐week period. We consider the difference acceptable taken into account the CBCT image resolutions of 0.5 mm, and the reproducibility in determining the center of markers being be up to 0.13 mm. Chung et al. assessed 15 landmarks on a CIRS 605 head phantom and reported a similar mean 3D deviation of 0.5 ± 0.2 mm between Frame‐based and CBCT‐based coordinates — with a maximum up to 0.8 mm.^9^ We plan to perform this CBCT‐based vs. Frame‐based stereotactic space coordinates test on a semi‐annual basis, with a tolerance limit of 1 mm.

**Table 2:** Coordinates of each marker in Frame‐based stereotactic space and CBCT‐based stereotactic space and the differences between each definition as established in the agreement of CBCT‐based with Frame‐based stereotactic space test. The magnitude of vector difference is calculated as $r=\sqrt{{\left(x_{\mathrm{Frame}}-x_{\mathrm{CBCT}}\right)}^{2}+{\left(y_{\mathrm{Frame}}-y_{\mathrm{CBCT}}\right)}^{2}+{\left(z_{\mathrm{Frame}}-z_{\mathrm{CBCT}}\right)}^{2}}$

					Displacement (mm) in			
Marker ID	Coordinates definition	*x*	*y*	*z*	*x*	*Y*	*z*	*r (mm)*
BB1	Frame‐based	78.2	141.4	68.2	0.4	0	‐0.4	0.57
	CBCT‐based	77.8	141.4	68.6				
BB2	Frame‐based	101	107.1	83.2	0.3	0	‐0.3	0.42
	CBCT‐based	100.7	107.1	83.5				
BB3	Frame‐based	120	78	97.4	0.3	−0.1	0.1	0.33
	CBCT‐based	119.7	78.1	97.3				
BB4	Frame‐based	136.8	53.1	109.6	0.3	−0.3	0.3	0.52
	CBCT‐based	136.5	53.4	109.3				
BB5	Frame‐based	154.1	24.5	122.8	0.3	−0.3	0.5	0.66
	CBCT‐based	153.8	24.8	122.3				

### 3.D Accuracy tests results of IFMM system, registration algorithm, and delivery‐after‐shift

Table 3 summarizes our results for the accuracy tests of IFMM system, registration algorithm, and delivery‐after‐shift. For the IFMM system, the instantaneous reading value can fluctuate by up to ±0.1 mm while tracking a stationary object, and we found the accuracy of the system to be within that range. The maximum differences we found were 0.23 mm when assessing only the translational IFMM accuracy (for a 5‐mm shift test in *x* direction), and −0.27 mm when adding the 1 degree rotation to the combination of translational large shift (5 mm in *x* and *y* directions, and 20 mm in *z* direction). Dorenlot and Champudry reported IFMM accuracy of 0.01 mm with a maximum error of 0.05 mm when assessing the translation accuracy with a micrometer screw.^10^

**Table 3:** Summary results of accuracy tests of the Intra‐fraction Motion Management system (IFMM), registration algorithm, and delivery‐after‐shift to a film. The results for IFMM and registration (3D stage method) and the shot measurements are averaged over small (2 mm in each of *x*,* y*, and *z* direction) and large (5 mm in *x* and *y* directions, 20 mm in *z* direction, and 1 degree rotation along *y*) shift test scenarios. The difference is calculated as measured value minus expected value

Test		Average difference ± SD (mm)	[Range] (mm)
IFMM accuracy	(3D stage method)	0.00 ± 0.12	[−0.27, 0.23]
	(Transformation matrix method)	0.08 ± 0.08	[−0.07, 0.15]
Registration accuracy	(3D stage method)	0.03 ± 0.06	[−0.06, 0.23]
	(Transformation matrix method)	−0.01 ± 0.13	[−0.48, 0.21]
Delivery‐after‐shift accuracy		0.24 ± 0.09	[0.08, 0.42]

The registration algorithm test also showed accuracy in the order of 0.1 mm, with a maximum of 0.23 mm difference in the *z* direction. The agreement in the rotational shift was within 0.01 ± 0.01 degrees with a maximum of 0.03 degree.

We found the transformation matrix method we used to test the accuracies of the registration algorithm and the IFMM system to be a useful test with submillimeter accuracy. This method can easily be applied in clinical settings to check the registration algorithm, provided that coordinates of a landmark are identified in both the reference CBCT and setup CBCT in the treatment console. As described by Wright et al., the IFMM system can be checked in a clinical setting by utilizing a second setup CBCT to calculate the 3D vector of a point close to the nose IR reflector; and the calculated vector would corresponds to the IFMM displacement magnitude.^3^ One limitation of the transformation matrix method is that it is based on the accurate reading of the coordinates in Icon's CBCT images, which has a voxel size of 0.5 mm.

Our accuracy test results for the film test was close to the values reported by the manufacturer,^11^ indicating submillimeter accuracy of the Icon to deliver accurate radiation shots based on its image guidance system. Zeverino et al. carried out an experiment by delivering 16‐mm shot sets at the center of a Ball Cube phantom placed in CBCT LSC center, with embedded orthogonal films, after shifting (15 mm lateral, 10 mm vertical, and 20 mm longitudinal) and reported a maximum CBCT correction (i.e., coregistration error) of 0.4 mm. Their experiment also indicated CBCT isocenter to be within 0.2 mm of the radiological focus.^2^

In clinical practice, one ambiguity the user faces is that the system‐proposed translational corrections are not visible to the user in terms of updated couch positions after the co‐registration of setup CBCT with the reference CBCT to account for patient interfractional shifts, and thus the user is left to trust the system automatic application of these shifts without any verification. We feel that testing the unit's determination of shift and applying it correctly is an important routine QA needed to ensure the safe use of the system. We plan to perform these accuracy tests of IFMM, registration, and deliver‐after‐shift on annual bases. Taking into consideration the limitations of our testing tool and methodology, we set a 0.5 mm tolerance criterion for each test, and a 0.5 degree for the registration algorithm test.

### 3.E CBCT dose constancy test

Our CTDI_w_ results have been consistent for the commissioning and two annual dose measurements we had. For the low‐dose preset, we measured 2.3 ± 0.0 mGy (vs 2.5 mGy nominal), and for the high‐quality preset, we measured 5.7 ± 0.1 mGy (vs 6.3 mGy nominal). Our CTDI_w_ dose measurements are within 10% of nominal values. Zeverino et al. reported CTDI_w_ values closer to nominal (2.41 and 6.32 mGy for low/high presets, respectively),^2^ while Dorenlot and Champoudry reported values similar to ours (2.23 and 5.9 mGy low/high presets, respectively).^10^ According to the manufacturer, less than ±35% deviations from the nominal values of the Icon's x‐ray unit are expected, and the differences are dependent on the generator, tube output, and manufacturing of the unit covers.^5^ We have established our tolerance criteria for this test to be ±5% of our CTDI_w_ baselines, and plan to continue performing this test on an annual basis as recommended by AAPM Task Groups 142^7^ and 179.^8^

## 4 CONCLUSION

In this study, we combined the manufacturer‐required routine QA checks with additional user‐defined checks for a comprehensive and robust quality assurance program for the Gamma Knife Icon's image guidance system ensuring its safe use. We found our system performance to meet the manufacturer specifications and our set limits, and to be comparable to other reported values in the literature.

## CONFLICT OF INTEREST

The authors have no conflicts of interest to disclose related to the topic of this work.

## References

[1] AlDahlawi I , Prasad D , Podgorsak MB . Evaluation of stability of stereotactic space defined by cone‐beam CT for the Leksell Gamma Knife Icon. J Appl Clin Med Phys. 2017;18:67–72. 28419781 10.1002/acm2.12073 PMC5689865

[2] Zeverino M , Jaccard M , Patin D , et al. Commissioning of the Leksell Gamma Knife^®^ Icon™. Med Phys. 2017;44:355–363. 28133748 10.1002/mp.12052

[3] Wright G , Harrold N , Hatfield P , Bownes P . Validity of the use of nose tip motion as a surrogate for intracranial motion in mask‐fixated frameless Gamma Knife^®^ Icon™ therapy. J Radiosurg SBRT. 2017;4:289–301. 29296453 PMC5658824

[4] Geometric Quality Assurance for Leksell Gamma Knife^®^ Icon™ (White Paper). Art. No. 1518416.01.

[5] Leksell Gamma Knife^®^ Icon™ Instructions for Use (Doc. ID: 1505194 Rev. 01). Stockholm, Sweden; 2015.

[6] The Measurement, Reporting, and Management of Radiation Dose in CT. Report of AAPM Task Group 23 of the Diagnostic Imaging Council CT Committee. American Association of Physicists in Medicine; 2008.

[7] Klein EE , Hanley J , Bayouth J , et al. Task Group 142 report: quality assurance of medical accelerators. Med Phys. 2009;36:4197–4212. 19810494 10.1118/1.3190392

[8] Bissonnette J‐P , Balter PA , Dong L , et al. Quality assurance for image‐guided radiation therapy utilizing CT‐based technologies: a report of the AAPM TG‐179. Med Phys. 2012;39:1946–1963. 22482616 10.1118/1.3690466

[9] Chung HT , Kim JH , Kim JW , et al. Assessment of image co‐registration accuracy for frameless gamma knife surgery. PLoS ONE. 2018;13:1–16. 10.1371/journal.pone.0193809 PMC5834193 29499061

[10] Dorenlot A , Champoudry J . End‐to‐end tests of the new Elekta gamma knife ICON. Phys Med. 2016;32:353.26818470

[11] Position accuracy analysis of the stereotactic reference defined by the CBCT on Leksell Gamma Knife^®^ Icon™ (White Paper); 2015. Art. No. 1509392.03.

